# A review of *Panelus* occurring in the Korean Peninsula (Coleoptera, Scarabaeidae, Scarabaeinae) with descriptions of two new species

**DOI:** 10.3897/zookeys.1292.200337

**Published:** 2026-09-16

**Authors:** Jaeil Shim, Kee-Jeong Ahn, Jeong-Hun Song

**Affiliations:** 1 Department of Agricultural Biology, National Institute of Agricultural Sciences, Wanju 55365, Republic of Korea Department of Agricultural Biology, National Institute of Agricultural Sciences Wanju Republic of Korea; 2 Department of Biology, Chungnam National University, Daejeon 34134, Republic of Korea Department of Biology, Chungnam National University Daejeon Republic of Korea; 3 Planning and Coordination Division, National Institute of Agricultural Sciences, Wanju 55365, Republic of Korea Planning and Coordination Division, National Institute of Agricultural Sciences Wanju Republic of Korea

**Keywords:** *COI*, dung beetle, Korean Peninsula, *
Panelus
*, taxonomy

## Abstract

A taxonomic review of Korean *Panelus* Lewis is presented based on morphological and molecular characters (*COI*). Detailed comparison of morphological characters and molecular analyses using genetic divergence and gene-tree monophyly for species delimitation support the description of two new species, Panelus (Panelus) virginalis Shim & Song, **sp. nov**. and Panelus (Panelus) jinilli Shim & Song, **sp. nov**. Intraspecific genetic divergence of *COI* gene using uncorrected p-distance ranged from 0.1% to 2.1% among the four *Panelus* species examined, while interspecific divergence ranged from 5.5% to 15.2% among all four species, including the two new Korean species and the two morphologically most similar Japanese species [P. (P.) parvulus (Waterhouse) and P. (P.) ovatus Nomura]. Morphological characters of the Asian species including two new species are compared. The new species are described with illustrations of diagnostic characters, and a key to the East Asian species of *Panelus* is presented.

## Introduction

The genus *Panelus* Lewis, date contains 39 species in two subgenera and occurs in the Palaearctic and Oriental regions (Ochi et al. 1998; Kawahara et al. 2007; Masumoto et al. 2011; Schoolmeesters 2026). Panelus (Panelus) parvulus, described from Japan by Waterhouse (1874), was first recorded in Korea by Kim (2004) and remained the only species of the genus *Panelus* known from the Korean Peninsula prior to the present study. Subsequently, Kim (2012) provided illustrations of the aedeagus of this species, but we noticed that the form and structures of Korean specimens differ from those of P. (P.) parvulus as illustrated by Kawai et al. (2008). This led us to study the species delimitation of these *Panelus* species in more detail based on morphological and molecular characters (*COI*).

Among the Korean species of *Panelus*, two species were recognized that were morphologically similar to the Japanese Panelus (Panelus) parvulus (Waterhouse) and P. (P.) ovatus Nomura, respectively. When compared with the aforementioned Japanese species, each Korean species showed morphological differences in punctation and morphology of aedeagus and the spermatheca. We further examined the species boundaries of these morphologically similar Korean and Japanese *Panelus* species based on genetic divergence and gene-tree monophyly using *COI* sequences and provide a key to the *Panelus* species distributed in Japan and Taiwan that are geographically closest to and morphologically most similar to the Korean new species (Nomura 1973a, 1973b; Ochi et al. 1998; Kawahara et al. 2007; Masumoto et al. 2011).

## Materials and methods

The morphological terminology used follows Vaz de Mello et al. (2011) and Gunter and Weir (2019). A photograph of the holotype of Panelus (Panelus) parvulus was provided by the Natural History Museum (**NHMUK**), London, UK. Depository of specimens examined is as follows: Chungnam National University Insect Collection (**CNUIC**), Daejeon, Korea; National Agricultural Science Insect Collection (**NASIC**), Wanju, Korea; Sungshin Women’s University Insect Collection (**SWUIC**) Seoul, Korea; National Institute of Biological Resources (**NIBR**), Incheon, Korea.

Specimens were examined under an Olympus SZX–16 stereomicroscope fitted with an Olympus DP–71 digital camera (Olympus Corporation, Tokyo, Japan). Habitus and structural images were obtained using a Canon EOS 5D DSLR camera, equipped with a Canon MP-E 65 mm f/2.8 1–5× macro lens. Dissected male genitalia and spermatheca were taken using an image processing system LEICA DMC2900 digital camera mounted on a LEICA M205 C microscope (Leica Geosystems AG). The images were merged using image stacking software, either CombineZP or Helicon Focus 6.2.2. and images were postprocessed using Adobe Photoshop CC 2020 (Adobe Systems, San Jose, CA, USA).

For molecular analysis, eleven Korean and Japanese *Panelus* specimens were used for DNA extraction (Table 1) and partial mitochondrial *COI* (658 bp) sequences were analyzed for comparison. The primer set followed Folmer et al. (1994), using LCO1490 (forward: 5’-GGTCAACAAATCATAAAGATATTGG-3’) and HCO2198 (reverse: 5’-TAAACTTCAGGGTGACCAAAAAATCA-3’), and the amplification profile was the same as that of Shim et al. (2021). Phylogenetic analyses were conducted using the maximum likelihood method implemented in PhyML 3.3 (Guindon et al. 2010) under the default settings in Geneious Prime 2025.0.3 (www.geneious.com). A neighbor-joining (NJ) analysis using the Kimura 2-parameter (K2P) model (Kimura 1980) and a parsimony (MP) analysis were performed in MEGA 12 (Kumar et al. 2024). Bootstrap support values for each node were evaluated via MEGA 12 with 1000 replicates. Genetic distances at intra- and interspecific comparisons, were computed using the uncorrected pairwise distance method as described by Srivathsan and Meier (2012).

**Table 1.** List of species, voucher numbers, and GenBank accession numbers used in this study. Dashes indicate unavailable data. Asterisks indicate new sequences.

**No**	**Species**	**Voucher no**.	**GenBank accession no**.	**Remarks**
1	*Panelus jinilli* sp. nov.	23676	*PZ022031	**Holotype**, NASIC
2	*P. jinilli* sp. nov.	23704	*PZ022032	**Paratype**, NASIC
3	*P. jinilli* sp. nov.	23705	*PZ022033	**Paratype**, NASIC
4	*P. ovatus*	23946	*PZ022034	Voucher specimen, NASIC
5	*P. virginalis* sp. nov.	23739-1	*PZ022035	**Holotype**, NASIC
6	*P. virginalis* sp. nov.	23739-2	*PZ022036	**Paratype**, NASIC
7	*P. parvulus*	23717	*PZ022037	Voucher specimen, NASIC
8	*P. parvulus*	23716	*PZ022038	Voucher specimen, NASIC
9	*P. parvulus*	23718	*PZ022039	Voucher specimen, NASIC
10	*P. parvulus*	23719	*PZ022040	Voucher specimen, NASIC
11	*P. parvulus*	23720	*PZ022041	Voucher specimen, NASIC
12	*Copris tripartitus*	-	MG701094	Out group, NCBI data
13	*Sisyphus schaefferi*	-	KM452277	Out group, NCBI data

For comparative morphology, we used one specimen of P. (P.) ovatus and eight specimens of P. (P.) parvulus deposited in NASIC, together with a high-resolution photograph of the NHMUK holotype of *Temnoplectron parvulum* [= Panelus (Panelus) parvulus (Waterhouse, 1874)]; label data for the examined material are provided in Suppl. material 1. For the molecular analysis, two Scarabaeinae species were used as outgroups (NCBI accession nos. KM452277, MG701094), and 11 new *COI* sequences (658 bp) from four *Panelus* species were generated and deposited in GenBank (PZ022031–PZ022041; Table 1).

## Taxonomy

### Genus Panelus

Taxon classificationAnimaliaColeopteraScarabaeidae

Lewis, 1895

950E6640-77C9-557F-94E8-44DC72D19530

#### Type species.

*Temnoplectron parvulum* Waterhouse, 1874 [= Panelus (Panelus) parvulus (Waterhouse, 1874)].

#### Diagnosis.

Member of the genus *Panelus* can be recognized by the combination of the following characters: body very small and oval; body brown to black; clypeal margin with two to four clypeal teeth; nine antennomeres present; pronotum with distinctly angled posterior margin; mesoventrite boomerang shaped; metaventrital process elongate, sub–triangular to pentagonal; protibiae with three teeth and serrations and apical digit; protarsi short; meso- to metatibiae slightly curved, without distinct spines, slightly curved; parameres of aedeagus asymmetrical (Lewis 1895; Kawahara et al. 2007; Masumoto et al. 2011; Ochi and Kon 2016).

##### Key to *Panelus* species in Korea, Japan, and Taiwan (modified from Kawahara et al. 2007; Masumoto et al. 2011)

**Table:** 

1	Metaventrite > 8.5× as long as mesoventrite; two to three fine serrations present between 1^st^ and 2^nd^ protibial teeth	**2**
–	Metaventrite < 7.5× as long as mesoventrite; four to five fine serrations present between 1^st^ and 2^nd^ protibial teeth	**3**
2	Metaventrite shorter than 2.2× the distance between the lateral angles of the metaventrital medial lobe	**Panelus (Panelus) manmiaoae Masumoto, Ochi & Tsai, 2011** [Taiwan]
–	Metaventrite longer than 2.5× the distance between the lateral angles of the metaventrital medial lobe	**4**
3	Elytra bicolored, reddish brown in humeral area, remaining area darker than humeral area	**P. (P.) wangi Masumoto, Ochi & Tsai, 2011** [Taiwan]
–	Elytra unicolored	**5**
4	Body reddish brown; metaventrite nearly 2.9× the distance between the lateral angles of the medial lobe	**P. (P.) crenatus Nomura, 1973** [Taiwan]
–	Body brown to dark brown; metaventrite shorter than 2.75× the distance between the lateral angles of the medial lobe	**6**
5	Elytra > 2.2× as long as pronotum	**7**
–	Elytra < 2.1× as long as pronotum	**8**
6	Mesoventrite with indistinct micropunctures (Fig. 4C); elytra ~ 1.18× as wide as long (Fig. 4A)	**P. (P.) ovatus Nomura, 1973** [Japan]
–	Mesoventrite with distinct micropunctures (Fig. 4D); elytra ~ 1.30× as wide as long	**P. (P.) jinilli sp. nov**. [Korea]
7	Pronotum and pygidium punctation indistinct, rather sparsely and shallowly punctate	**P. (P.) rufulus Nomura, 1973** [Japan]
–	Pronotum and pygidium punctation distinct, coarsely and densely punctate	**P. (P.) maedai Nomura, 1973** [Taiwan]
8	Elytra widest at the middle	**P. (P.) kubotai Kawahara, Inagaki & Ochi, 2007** [Japan]
–	Elytra widest at the anterior one-third	**9**
9	Body reddish brown to dark brown; mesoventrite punctures indistinct, surface smooth (Fig. 3C); spermatheca C-shaped, medial area inflexed, both distal area rather sharp (Fig. 3L)	**P. (P.) parvulus (Waterhouse, 1874)** [Japan]
–	Body reddish brown; mesoventrite punctures distinct, coarse and circle shaped (Fig. 2C, D); spermatheca L-shaped, curved hook shaped, distal area clavate shape (Fig. 2H)	**P. (P.) virginalis sp. nov**. [Korea]

##### Species descriptions

### Panelus (Panelus) jinilli

Taxon classificationAnimaliaColeopteraScarabaeidae

Shim & Song sp. nov.

D2419E5C-2A24-52D7-8A69-4A1628106598

https://zoobank.org/93B64B01-D039-49EC-B10F-1064511519A9

Fig. 1

#### Type material.

***Holotype***: South Korea • ♂; Jeollabuk-do Prov., Wanju-gun, Gosan-myeon, Seongjae-ri, 35°56'32.26"N, 127°13'14.19"E, 160 m, 7 Sep 2024, SY Hong, deciduous, ex. dung baited pitfall trap (cow); HOLOTYPE, Panelus (Panelus) jinilli sp. nov. Shim & Song, Desig. J. Shim & J.-H. Song 2026 (GenBank: PZ022031) (NASIC). ***Paratypes***: South Korea • 1♂; same data as for holotype (GenBank: PZ022032) (NASIC) • 2♀; Chungcheongnam-do Prov., Taean-gun, Sowon-myeon, Uihang-ri, Baekripo beach, 31.VII–7.IX.2013, CNUIC Col., ex. FIT (NASIC) • 1♂; Chungcheongnam-do Prov., Taean-gun, Sowon-myeon, Uihang-ri, Baekripo beach, 31.VII–7.IX.2013, CNUIC Col., ex. FIT (NIBR no. NIBRIN0001103794) • 1 ex; Chungcheongnam-do Prov., Temple Janggoksa, Mt. Chilgapsan, Janggok-ri, Daechi-myeon, Cheongyang-gun, 3–14.VI.2000, K.H. Jung, M.J. Lee, J.H. Song, ex. FIT (CNUIC) • 1 ♀; same data as for holotype (GenBank: PZ022033) (NASIC) • 1 ex; Jeollanam-do Prov., Jangseong-gun, Bugi-myeon, Jukcheong-ri, 35°26'25.42"N, 126°45'10.66"E, 318 m, 12 Nov 2024, SY Hong, ex dung baited pitfall trap (cat); PARATYPE, Panelus (Panelus) jinilli sp. nov. Shim & Song, Desig. J. Shim & J.-H. Song 2026 (NASIC).

**Figure 1.**
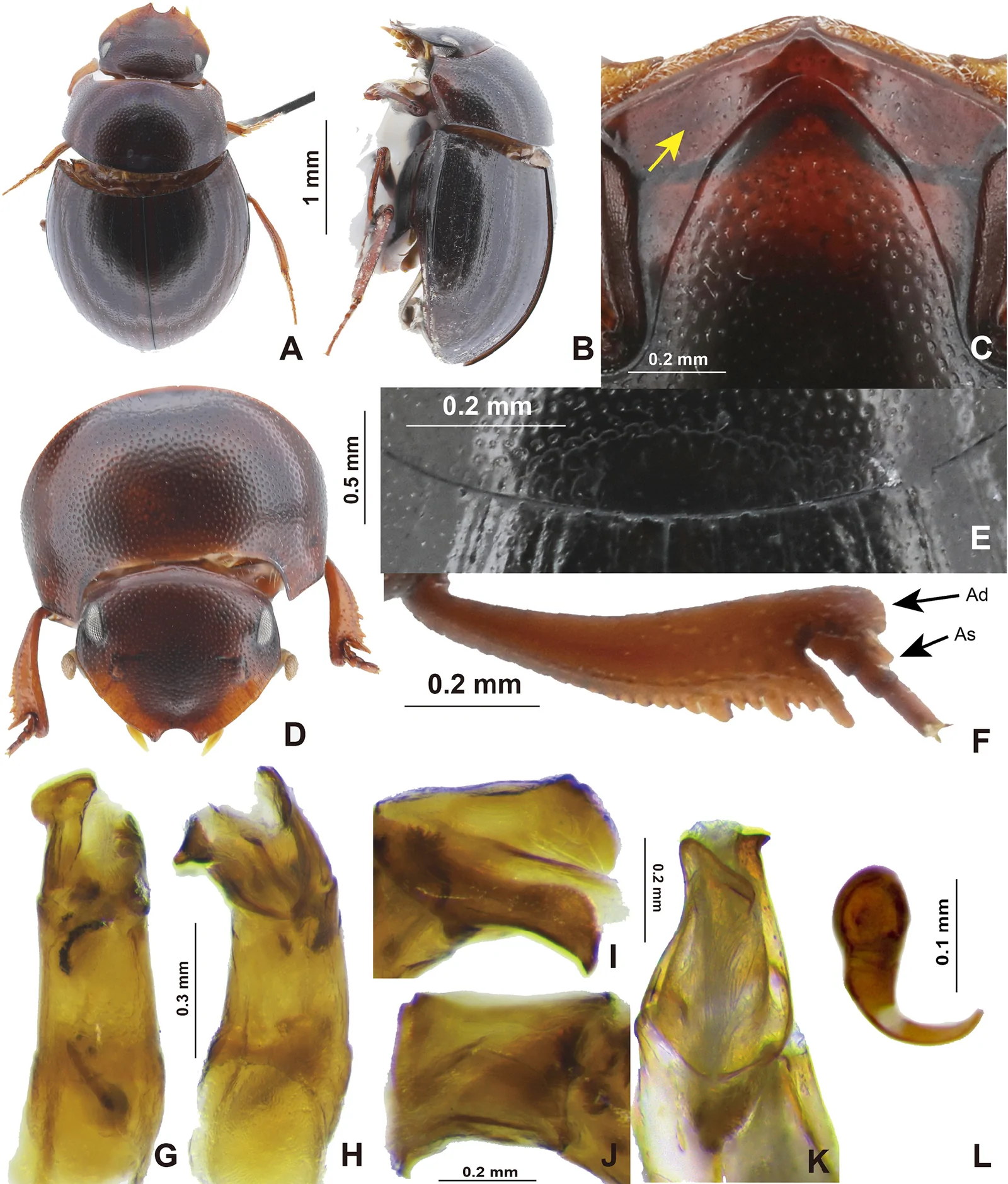
Panelus (Panelus) jinilli sp. nov. **A**. Male, dorsal aspect; **B**. Male, lateral aspect; **C**. Mesoventrite punctation; **D**. Head and pronotum; **E**. Pronotum posterior margin; **F**. Protibia; **G**. Male genitalia, dorsal aspect; **H**. Male genitalia, ventral aspect; **I**. Parameres, lateral aspect (left); **J**. Parameres, lateral aspect (right); **K**. Parameres, dorsal aspect; **L**. Spermatheca. Yellow arrow = punctation of mesoventrite. Abbreviations: Ad = apical digit; As = apical spur.

#### Diagnosis.

Panelus (Panelus) jinilli sp. nov. can be recognized by the combination of the following characters: body generally brown to dark brown (Fig. 1A); elytra 1.2× wider than pronotum; mesoventrite very narrowed and surface with micropunctures (Fig. 1C); median length of the metaventrite 9.5× longer than mesoventrite (Fig. 1C); between 1^st^ and 2^nd^ protibial teeth with two to three fine serrations (Fig. 1F).

#### Description.

***Total body length***: 1.9–2.6 mm. **Male**. Very small sized beetles. Body oval in shape. ***Coloration*** (Fig. 1A, B): Body dark brown, surface shiny.

***Head*** (Fig. 1A, D): 1.26× wider than long; sub-pentagonal shaped, dorsal surface with microreticulation, densely distributed setigerous micropunctures (setae very thin at 150×). Clypeal margin concave at the middle; two sharp clypeal teeth weakly upturned, its dorsal surface with short setae. Clypeogenal suture traceable, its basal area with short and shallow excavation, apical area of clypeogenal suture area with blunt teeth (angulate). Genal angle, widely round. Eye size moderate and half-obovate shape in dorsal view, sub-trapezoidal shape in ventral view. Ocular canthus divided compound eye, frequently with very narrow interocular space. Gula anterior area divided by wide U-shaped suture, anterior surface rugose.

***Pronotum*** (Fig. 1A, B, D, E): Pronotum disc sub-rectangular, moderately convex, widest in posterior angle, very slightly narrowed to anteriorly; pronotum nearly 1.8× wider than long. Pronotum maximum width as long as elytra length; elytra width nearly 1.2× as wide as pronotum. Pronotum anterior angle acute, lateral margin sinuate, arcuate at the middle in lateral view; posterior angle obtuse. Pronotum dorsal surface very densely punctate; punctation along the posterior margin forming a row, coarser than remains; medio-posterior margin surface (Fig. 1E) with fused punctures (elliptical shape, nearly 1/3 length of posterior margin).

***Pterothorax*** (Fig. 1C): Mesoventrite with moderately distribute micropunctures; mesoventrite narrowed medially; median lengths of metaventrite 9.5× as long as mesoventrite. Metaventrite medial lobe adjacent to mesoventrite swelling, medial lobe elongated sub-pentagonal shape, its lateral margin very widely angled at the anterior 1/3 area, surface punctures distinct, moderately larger to laterad; lateral surface with circle to oval shaped punctation.

***Elytra*** (Fig. 1A, B): Moderately convex and widened in anterior 1/3 area; elytra 1.1–1.2× wider than long. Elytra with nine striae and nine intervals clearly visible. Striae thin and shallow; 7^th^ stria shortened before posterior 1/3 area; 8^th^ stria soared and keel-like, its fused with 9^th^ stria in posterior 1/3 area; strial punctures rather distinctly indenting margins of intervals, distinctly larger than punctures of intervals. Intervals shiny; its anterior margin with a blunt tubercle; intervals rather densely punctate with setigerous micropunctures, 1^st^ interval with three rows of punctures, punctures of remain intervals forming five rows; 9^th^ interval more elevated than remaining intervals. Elytral margins border with coarse punctures.

***Legs*** (Fig. 1A, B, F): Protibiae (Fig. 1F) slightly curved inwardly, moderately widened apically; protibiae anterior margin deeply concave at the middle; apical digit well developed and apical area round; outer margin with three large tibial teeth, between tibial teeth with two or three fine denticles. Meso- and metatibiae slightly widened to apically; metatibiae lateral apex forming crenulated keel. Meso- and metatarsi of 2^nd^–4^th^ tarsomeres subequal in size. Tarsal claws minute and finely toothed (triangular tubercle).

***Abdomen***. Abdominal sternites shiny, anterior margin of 1^st^–4^th^ sternites with a row of fine punctures; 5^th^ sternite with regularly distributed two rows of punctures; 6^th^ sternite with irregularly distributed and forming five or six rows of punctures; 6^th^ sternite postero-medial margin widely concave at the middle. Pygidium with similar structure to 6^th^ sternite, but more densely punctate.

***Aedeagus*** (Fig. 1G–K): Parameres (Fig. 1I, J) distinctly asymmetrical; left paramere (Fig. 1I) narrow and trapezoidal shape in lateral view, its ventral apex sharp and slightly curved outwardly (Fig. 1K); right paramere (Fig. 1J, K) wide and its covered left paramere, outer margin distinctly concave, apical margin obliquely truncated, apical apex bilobed in lateral view (Fig. 1I). Parameres clearly shorter than phallobase (Fig. 1G, H).

**Female**. Very similar to male, with following differences. ***Abdomen***: Posterior margin of 6^th^ abdominal sternite transverse (longer than male). ***Spermatheca*** (Fig. 1L). Strongly curved comma-shaped.

#### Distribution.

(Fig. 5) South Korea (type locality: Seongjae-ri, Gosan-myeon, Wanju-gun, Jeollabuk-do).

#### Etymology.

The specific epithet jinilli is dedicated to the late Prof. Jin-Ill Kim of Sungshin Women’s University (Seoul, Korea) in recognition of his significant contributions to Korean entomology and his continuous support for research on Scarabaeoidea.

#### Remarks.

Panelus (Panelus) jinilli sp. nov. was previously identified as P. (P.) parvulus (Waterhouse) by Kim (2012). However, P. (P.) parvulus can be readily distinguished from P. (P.) jinilli sp. nov. by the following combination of characters (Fig. 3): pronotum medio-posterior margin fused-punctures area nearly 1/6–1/5 length of posterior margin (Fig. 3E); the mesoventrite punctures extremely small and indistinct (Fig. 3C); median length of metaventrite 5.6–5.9× as long as mesoventrite; medial lobe of metaventrite angled at the anterior 1/4 area of lateral margin (Fig. 3C); between 1^st^ and 2^nd^ protibial teeth with four or five fine serrations (Fig. 3F); male right paramere with dorsally curved structures (Fig. 3I), apical structure of left paramere strongly curved downward (Fig. 3J); spermatheca (Fig. 3L) C-shaped, with an inflexed medial area, rather sharp distal area.

Panelus (Panelus) jinilli sp. nov. is most similar to the Japanese P. (P.) ovatus Nomura. This species is also similar in internal characters, including the spermatheca (Fig. 4E, F), but has a narrower body and sparser, less distinct punctures on the pronotum, elytra and mesoventrite (Fig. 4A–D).

#### Biological note.

Panelus (Panelus) jinilli sp. nov. was primarily collected using flight interception traps (F.I.T.) and was also attracted to bait consisting of various types of animal dung, including that of leopard cat and cow. Additional surveys confirmed that the species was also collected from deer dung and decaying feathers. Most specimens were obtained from low mountainous areas at elevations of approximately 200 m above sea level.

### Panelus (Panelus) virginalis

Taxon classificationAnimaliaColeopteraScarabaeidae

Shim & Song sp. nov.

802478E5-B18F-5DFA-BF83-64BDEC4DFD58

https://zoobank.org/D1B1129C-271D-476A-BBD3-54878C063042

Fig. 2

#### Type material.

***Holotype***: South Korea • 1♀; Jeollanam-do Prov., Gurye-gun, Ganjeon-myeon, Jungdae-ri, Hanjae valley, 29.V–11.VII.2024, J.B. Seung, ex. FIT; HOLOTYPE, Panelus (Panelus) virginalis sp. nov. Shim & Song, Desig. J. Shim & J.-H. Song 2026 (GenBank: PZ022035) (NASIC). ***Paratypes***: South Korea • 2♀; same data as for holotype (GenBank: PZ022036) (NASIC) • 1♀; Gangwon-do Prov., Yanggu-gun, Yanggu-eup, 38°07'00.5"N, 127°93'49.8"E, 14.VII–12.VIII.2003, S.J. Park, D.H. Lee, ex. FIT (NASIC) • 1♀; Chungcheongnam-do Prov., Yuseong-gu, Mt. Gyeryongsan, Geumsubong peak, 21.V–3.VI.2000, S.J. Park, M.S Kim, ex. FIT (CNUIC) • 17♀♀; Chungcheongnam-do Prov., Gongju-si, Banpo-myeon, Hakbong-ri, Mt. Gyeryongsan, Temple Gapsa, 3–14.VI.2000, S.J. Park, M.S. Kim, ex. FIT (CNUIC) • 44♀♀; Chungcheongnam-do Prov., Gongju-si, Banpo-myeon, Hakbong-ri, Mt. Gyeryongsan, Woosanbong peak, 10–24.VII.2000, S.J. Park, M.S Kim, ex. FIT (CNUIC) • 1♀; Chungcheongnam-do Prov., Yuseong-gu, Mt. Gyeryongsan, Geumsubong peak, 24.VII–2.VIII.2000, S.J. Park, M.S Kim, ex. FIT (CNUIC) • 1♀; Chungcheongnam-do Prov., Gongju-si, Banpo-myeon, Sangsin-ri, Mt. Gyeryongsan, Pagodas Nammaetap, 36°21'19"N, 127°13'21"E, 19–30.V.2006, S.I. Lee, T.K. Kim, ex. bait trap (CNUIC) • 18♀♀; Chungcheongnam-do Prov., Gongju-si, Banpo-myeon, Hakbong-ri, Mt. Gyeryongsan, Temple Gapsa, 36°22'03.2"N, 127°12'50"E, 31 V–18.VI.2004, K.J. Ahn, S.M. Choi, J.S. Park, ex. FIT (CNUIC) • 6♀♀; Chungcheongnam-do Prov., Gongju-si, Banpo-myeon, Sangsin-ri, Mt. Gyeryongsan, 36°22'03.2"N, 127°12'50"E, 31 V–18.VI.2004, S.M. Choi, J.S. Park, ex. FIT (CNUIC) • 1♀; Chungcheongnam-do Prov., Dongwol valley, Gongju-si, Banpo-myeon, Hakbong-ri, Mt. Gyeryongsan, 36°19'39"N, 127°15'46.7"E, VI 2004, S.M. Choi, J.S. Park, ex. FIT (CNUIC) • 78♀♀; Chungcheongnam-do Prov., Gongju-si, Banpo-myeon, Hakbong-ri, Mt. Gyeryongsan, 36°20'27.8"N, 127°15'11.5"E, 1–18.VI.2004, K.J. Ahn, S.M. Choi, J.S. Park, ex. FIT (CNUIC) • 1♀; Chungcheongnam-do Prov., Gongju-si, Banpo-myeon, Hakbong-ri, Mt. Gyeryongsan, Eunseon waterfall, 36°20'58.7"N, 127°12'41.3"E, 1–18.VI.2004, S.M. Choi, J.S. Park, ex. FIT (CNUIC) • 1♀; Chungcheongnam-do Prov., Gongju-si, Banpo-myeon, Hakbong-ri, Mt. Gyeryongsan, 36°20'27.8"N, 127°15'11.5"E, 1–18.VI.2004, K.J. Ahn, S.M. Choi, J. S. Park, ex. bait trap (CNUIC) • 7♀♀; Chungcheongnam-do Prov., Gongju-si, Banpo-myeon, Hakbong-ri, Mt. Gyeryongsan, Temple Donghaksa, 36°21'17.4"N, 127°14'55.7"E, 1–18.VI.2004, S.M. Choi, J.S. Park, ex. FIT (CNUIC) • 1♀; Chungcheongnam-do Prov., Gongju-si, Banpo-myeon, Hakbong-ri, Mt. Gyeryongsan, Temple Gapsa, 36°22'03.2"N, 127°12'50"E, 1–18.VI.2004, K.J. Ahn, S.M. Choi, J. S. Park, ex. FIT (CNUIC) • 47♀♀; Chungcheongnam-do Prov., Gongju-si, Banpo-myeon, Sangsin-ri, Mt. Gyeryongsan, Pagodas Nammaetap, 36°21'11.8"N, 127°13'20.8"E, 1–18.VI.2004, S.M. Choi, J.S. Park, ex. FIT (CNUIC) • 8♀♀; Jeollabuk-do Prov., Jeongeup-si, Naejang-dong, Mt. Naejangsan, Temple Naejangsa, Geumseon valley, 15–24.VI.2000, U.S. Hwang, H.J. Kim, ex. FIT (CNUIC) • 1♀; Jeollabuk-do Prov., Jeongeup-si, Naejang-dong, Mt. Naejangsan, Temple Naejangsa, Geumseon valley, 15–24.VI.2000, U.S. Hwang, H.J. Kim, ex. FIT (NIBR no. NIBRIN0001103795) • 4♀♀; Jeollabuk-do Prov., Sunchang-gun, Paldeok-myeon, Cheonggye-ri, Mt. Gangcheonsan, 13 X 2009, J.C. Jeong, ex. pit-fall trap (CNUIC) • 1♀; Jeollabuk-do Prov., Namwon-si, Valley of Baemsaggol, 30 VIII 2001 (SWUIC) • 3♀♀; Jeollabuk-do Prov., Mt. Jirisan, VIII 2003, Det. J.I. Kim (SWUIC) • 3♀♀; Jeollanam-do Prov., Jangseong-gun, Bukha-myeon, Mt. Naejangsan, Temple Baekyangsa, 15–24.VI.2000, U.S. Hwang, H.J. Kim, ex. FIT (CNUIC) • 1♀; Jeollanam-do Prov., Gwangju-si, Jisan-dong, Mt. Mudeongsan, Temple Wonhyosa, 20.VII–10.VIII.2000, U.S. Hwang, H.J. Kim and S.J. Park, ex. FIT (CNUIC) • 12♀♀; Jeollanam-do Prov., Boseong-gun, Bokrae-myeon, Mt. Dubongsan, 34°92'50.9"N, 127°09'70.1"E, 30.VI–29.VII.2003, S.J. Park, C.W. Shin, ex. FIT (CNUIC) • 2♀♀; Jeollanam-do Prov., Wando-gun, Peak Sangwangbong, 34°35'72.3"N, 126°71'31.1"E, 1 VII–29.VII.2003, S.J. Park, C.W. Shin, J.S. Park, ex. FIT (CNUIC) • 5♀♀; Jeollanam-do Prov., Haenam-gun, Samsan-myeon, Gurim-ri, Mt. Duryunsan, 34°28'4.0"N, 126°37'36.4"E, 350 m, 23–24.IX.2009, T.K. Kim, S.G. Lee, ex. pit-fall trap, shrimp bait (CNUIC) • 1♀; Jeollanam-do Prov., Jangseong-gun, Bugi-myeon, Jukcheong-ri, 35°26'25.42"N, 126°45'10.66"E, 318 m, 12 Nov 2024, SY Hong, ex dung baited pitfall trap (cat); PARATYPE, Panelus (Panelus) virginalis sp. nov. Shim & Song, Desig. J. Shim & J.-H. Song 2026 (NASIC).

**Figure 2.**
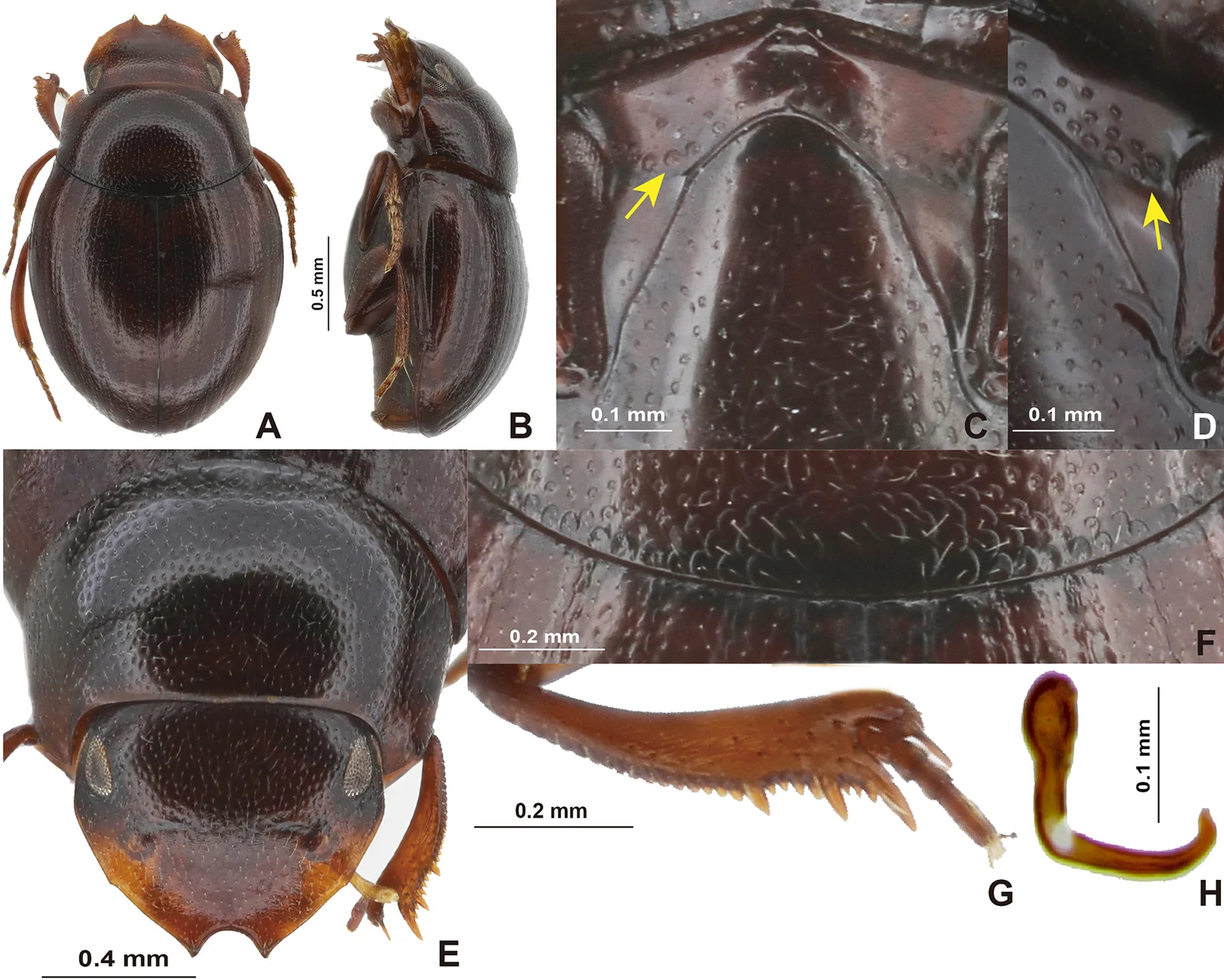
Panelus (Panelus) virginalis sp. nov. **A**. Female, dorsal aspect; **B**. Female, lateral aspect; **C, D**. Mesoventrite punctation; **E**. Head and pronotum; **F**. Pronotum posterior margin; **G**. Protibia; **H**. Spermatheca. Yellow arrows = punctation of mesoventrite.

#### Diagnosis.

Panelus (Panelus) virginalis sp. nov. can be recognized by the combination of the following characters: body reddish brown (Fig. 2A); pronotum rather narrowed, elytral width exceeding 1.27× wider than pronotum (Fig. 2A); mesoventrite somewhat narrowed and surface with distinct oval shaped punctures (Fig. 2C, D); median length of the metaventrite 7× longer than mesoventrite; between 1^st^ and 2^nd^ protibial teeth with four or five fine serrations (Fig. 2G); distinctly curved spermatheca (Fig. 2H).

#### Description.

***Total body length***: 1.7–2.4 mm. **Female**. Very small beetles. Body oval in shape. ***Coloration*** (Fig. 2A, B): Body reddish brown to brown, surface very shiny.

***Head*** (Fig. 2A, E): Head 1.23× wider than long; sub-pentagonal shaped, dorsal surface with microreticulation, densely distributed setigerous micropunctures (setae very thin at 150×). Clypeal margin concave at the middle, margin before clypeogenal suture nearly straight; two sharp clypeal teeth weakly upturned, its dorsal surface with short setae; clypeal border semi-translucent. Clypeogenal suture very weak, its basal area with short and shallow excavation, apical area of clypeogenal suture area with blunt teeth. Genal angle, widely round. Eye size moderate and half; obovate shape in dorsal view, sub-trapezoidal shape in ventral view. Ocular canthus divided compound eye, without interocular space. Gula anterior area divided by wide ⎵-shaped suture, anterior surface rugose.

***Pronotum*** (Fig. 2A, B, E, F): Pronotum disc sub-rectangular, moderately convex, widest in posterior angle, moderately narrowed to anteriorly; pronotum 1.7× wider than long. Pronotum maximum width distinctly shorter than elytra length; elytra width 1.27–1.36× as wide as pronotum. Pronotum anterior angle acute, lateral margin slightly sinuate; posterior angle obtuse. Pronotum dorsal surface very densely punctate with micropunctures; punctation along the posterior margin forming a row, 2–4× larger than remains; medio-posterior margin surface (Fig. 2F) with fused punctures (elliptical shape, nearly 1/6–1/5 length of posterior margin). Interior surface of hypomeron coarsely punctate.

***Pterothorax*** (Fig. 2C, D): Mesoventrite with rather sparsely distributed coarse punctures, its oval shape and smaller to anteriorly; mesoventrite narrowed medially; median lengths of metaventrite 7× as long as mesoventrite. Mesepimeron surface coarsely punctate. Metaventrite anterior margin of medial lobe round, its lateral margin very weakly sinuate (weakly prominent in anterior 1/4 and 3/4 areas), surface punctures distinct, moderately larger to laterad; lateral surface punctation elongate oval shape.

***Elytra*** (Fig. 2A, B): Moderately convex and widened in anterior 1/3 to medial area; elytra < 1.1× wider than long. Elytra with nine striae and nine intervals clearly visible. Striae thin and shallow; 7^th^ stria shortened before posterior 1/3 area; 8^th^ stria soared and keel-like, its fused with 9^th^ stria in posterior 1/3 area; strial punctures rather distinctly indenting margins of intervals, distinctly larger than punctures of intervals. Intervals shiny; its anterior margin with a blunt tubercle; intervals rather densely punctate with setigerous micropunctures, 1^st^ interval with three rows of punctures, punctures of remain intervals forming four to five rows; 9^th^ interval soared than remain intervals. Elytral margins border with coarse and shallow punctures.

***Legs*** (Fig. 2A, B, G): Protibiae (Fig. 2G) slightly curved inwardly, moderately widened apically; protibiae anterior margin deeply concave at the middle; apical digit well developed and apical area round, apical spur sharp; outer margin with three large tibial teeth, between 1^st^ and 2^nd^ tibial teeth with four to five fine teeth (serration), between 2^nd^ and 3^rd^ tibial teeth with two to three serrations. Meso- and metatibiae slightly widened to apically; metatibiae lateral apex forming crenulated keel. Meso-and metatarsi 2^nd^–4^th^ tarsomeres subequal in size. Tarsal claws minute, finely toothed.

***Abdomen***. Abdominal sternites shiny, anterior margin of 1^st^–4^th^ sternites with a row of fine punctures, its sternites medial area micro-rugose; 5^th^ sternite very narrow at the middle, surface with regularly distributed two rows of punctures; 6^th^ sternite with irregularly distributed and forming four to five rows of punctures, punctation slightly smaller to apically; 6^th^ sternite postero-medial margin transverse. Pygidium with similar structure to 6^th^ sternites, but more densely punctate.

***Spermatheca*** (Fig. 2H). L-shaped, elongate area curved hook shaped, comma head area 2× thicker than hook area.

#### Distribution.

(Fig. 5) South Korea (Type locality: Mt. Jirisan, Hanjae valley, Jungdae-ri, Ganjeon-myeon, Gurye-gun, Jeollanam-do).

#### Etymology.

The specific epithet ‘virginalis’ is derived from the Latin ‘virgo’, meaning “virgin” or “maiden”, and refers to the female-only composition of the material currently available for this species (> 260 individuals). The name is not intended to imply confirmed parthenogenesis, which remains to be tested with additional biological and molecular data.

#### Remarks.

**Figure 3.**
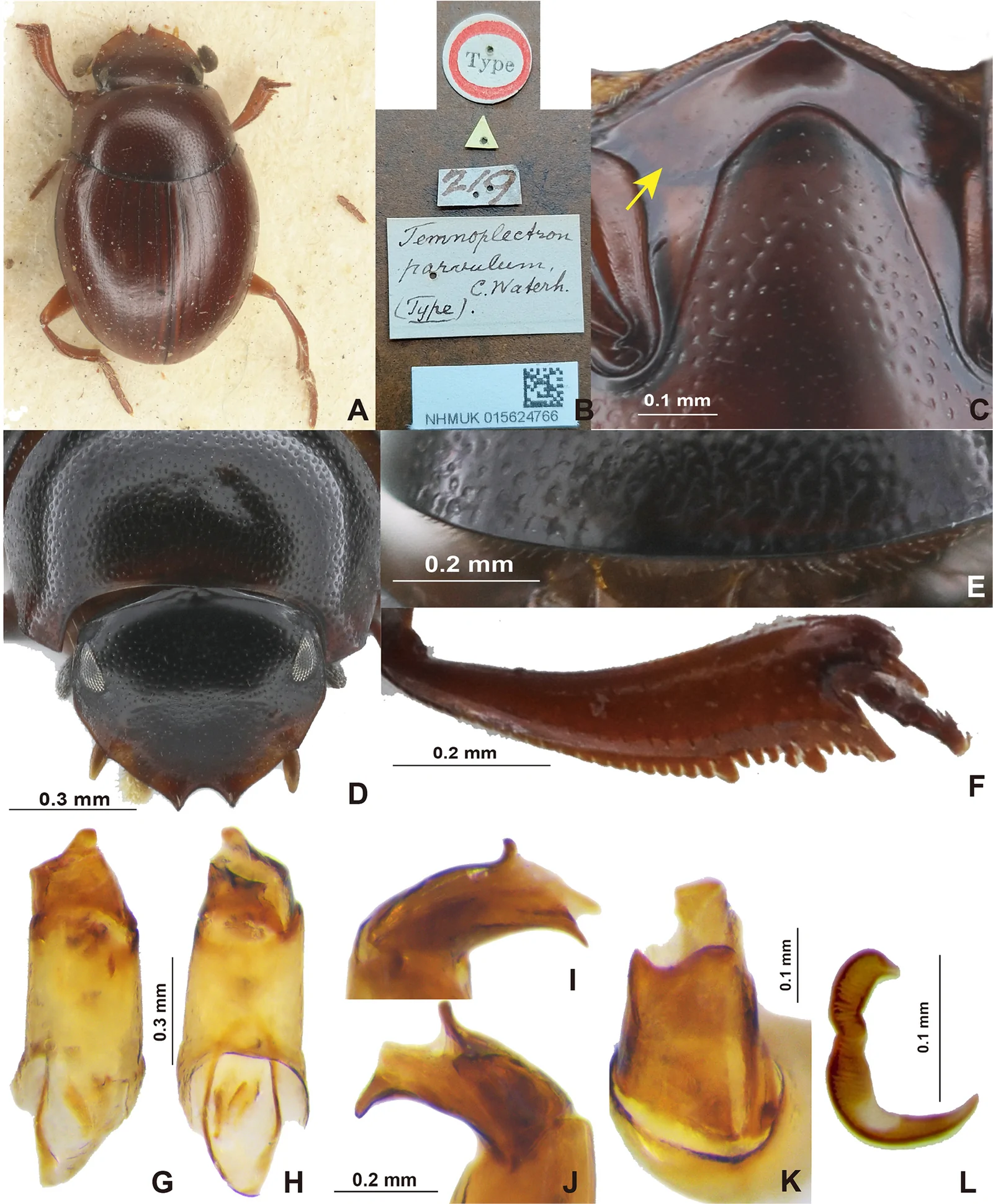
Panelus (Panelus) parvulus. **A**. Holotype, dorsal aspect; **B**. Holotype, label; **C**. Mesoventrite punctation; **D**. Head and pronotum; **E**. Pronotum posterior margin; **F**. Protibia; **G**. Male genitalia, dorsal aspect; **H**. Male genitalia, ventral aspect; **I**. Parameres, lateral aspect (left); **J**. Parameres, lateral aspect (right); **K**. Parameres, dorsal aspect; **L**. Spermatheca. Yellow arrow = punctation of mesoventrite. Holotype image (**A, B**; deposited in NHMUK) courtesy of D. Telnov (Natural History Museum, London).

Panelus (Panelus) virginalis sp. nov., like P. (P.) jinilli sp. nov., was previously identified as P. (P.) parvulus (Waterhouse) by Kim (2004, 2012) and is morphologically most similar to that species. However, unlike P. (P.) parvulus, P. (P.) virginalis sp. nov. possesses coarse punctures on the mesoventrite (Figs 2C, 2D, 3C). The two species also differ distinctly in the shape of the spermatheca (Figs 2H, 3L).

#### Biological note.

Most specimens of Panelus (Panelus) virginalis sp. nov. were collected using flight interception traps (F.I.T.). Approximately 5% of the specimens were collected using bait, including decaying meat, shrimp, and dead fish. Additional surveys confirmed the presence of this species in decaying feathers and deer dung, and it was more frequently collected from necrophagous baits than from coprophagous ones. Furthermore, P. (P.) virginalis sp. nov. was mainly collected in mountainous forest habitats at elevations above 300 m.

## Discussion

A distribution map (Fig. 5) shows that the two new Korean species and the two Japanese species examined in this study differ to some extent in their geographical distribution, despite their morphological similarities (Lewis 1895; Kawahara et al. 2007; Masumoto et al. 2011). However, Panelus (Panelus) jinilli sp. nov. and P. (P.) virginalis sp. nov., together with the Japanese P. (P.) parvulus, represent the northernmost known members of the genus *Panelus* (Kawai et al. 2008; Schoolmeesters 2026).

**Figure 4.**
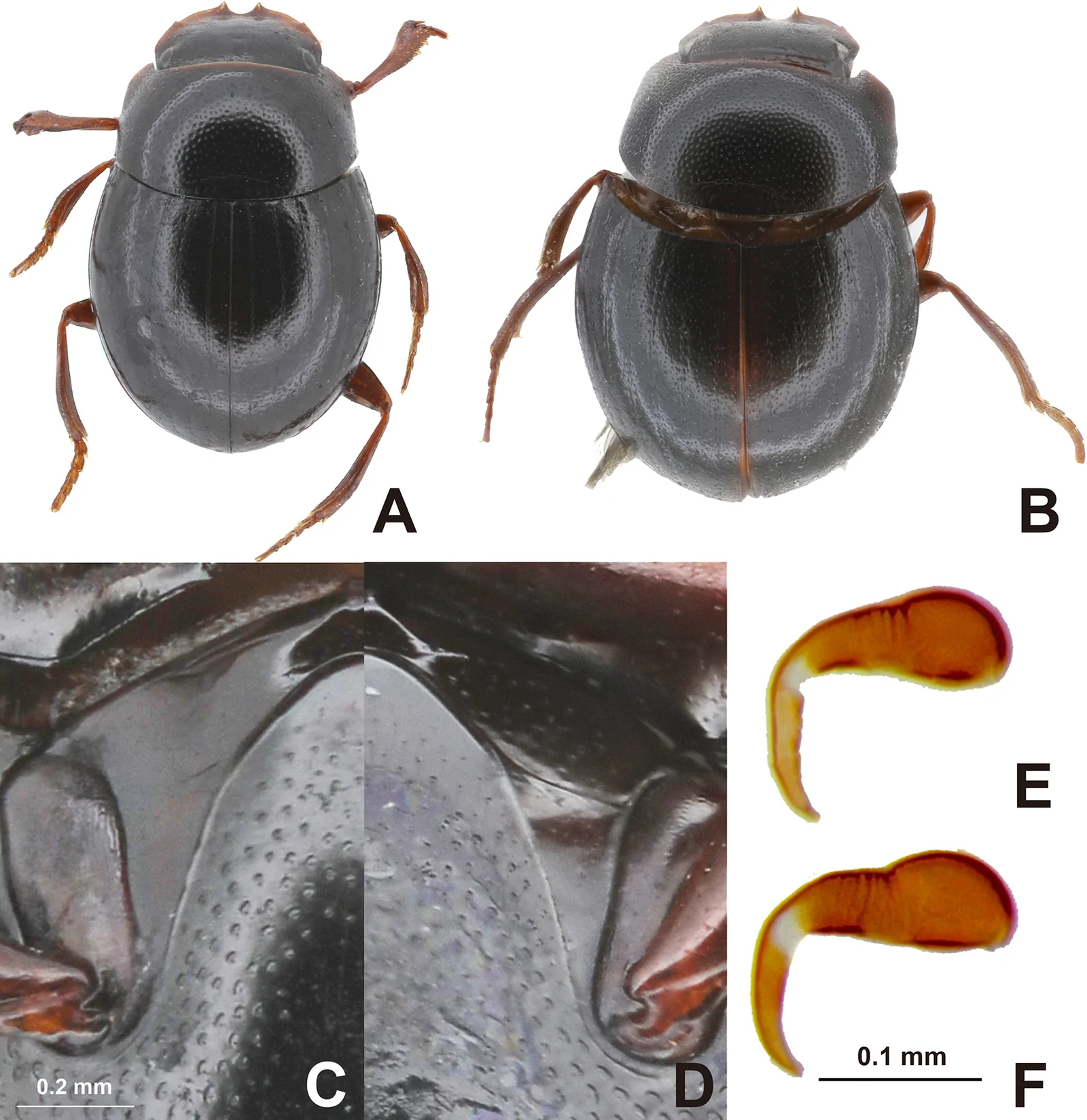
Morphological characters of Panelus (Panelus) ovatus (**A, C, E**) and Panelus (Panelus) jinilli sp. nov. (**B, D, F**). **A, B**. Adult, dorsal aspect; **C, D**. Mesoventrite punctation; **E, F**. Spermatheca.

Among morphologically similar *Panelus* species (*P. jinilli* sp. nov., *P. virginalis* sp. nov., *P. ovatus*, and *P. parvulus*), useful characters for species delimitation include body punctation, the shape of the meso-metaventrite, and the structures of the male genitalia and spermatheca (Figs 1, 2, 3, 4).

**Figure 5.**
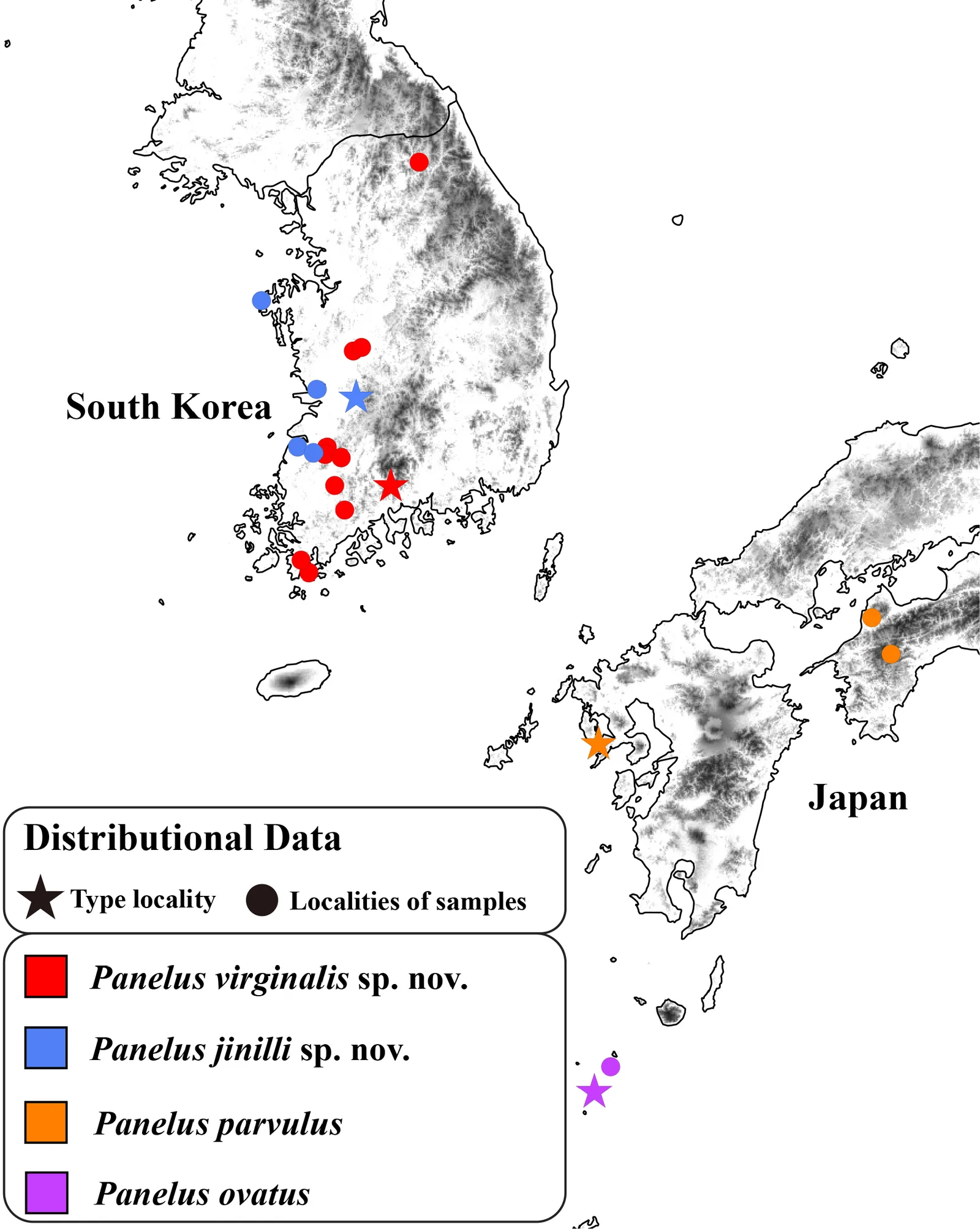
Distribution map of four *Panelus* species of Korea and Japan. Colored stars indicate type localities whereas colored circles represent examined localities of this study.

**Figure 6.**
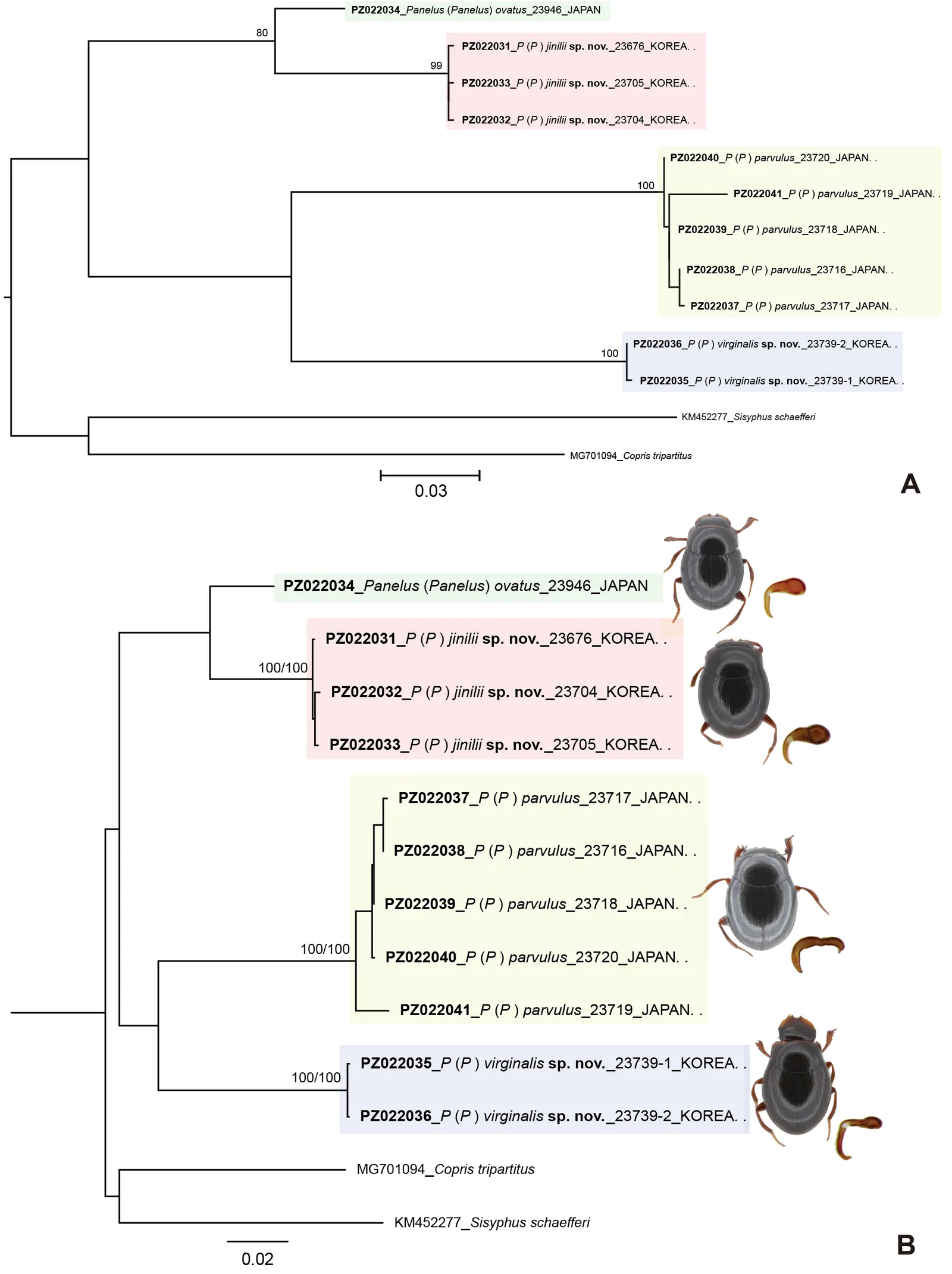
Gene tree of four *Panelus* spp. estimated by Maximum likelihood method and Neighbor-joining method using *COI* gene data. **A**. Maximum likelihood (ML) tree, Bootstrap support values > 50% are indicated above branches; **B**. Neighbor-joining (NJ) tree, bootstrap values (left) and Parsimony analysis bootstrap values (right). Scale bar indicates the expected number of substitutions per site. On the right, figures showing the dorsal habitus and spermatheca of each species are provided.

Pairwise distance data based on *COI* (658 bp) showed that intraspecific divergence within the four *Panelus* species ranged from 0–2.1% (mean 0.7%), indicating identical or very similar sequences. The smallest interspecific genetic distance was observed between *P. jinilli* sp. nov. and *P. ovatus*, ranging from 5.5–5.7%, whereas interspecific distances among the remaining species ranged from 12.6–15.2% (Table 2). In the maximum likelihood analysis (Fig. 6A), the two new Korean species were supported as valid independent species distinct from the two morphologically similar Japanese species. Likewise, in the Neighbor-Joining and parsimony analyses (not shown), all four species were also supported as separate lineages when morphological differences were considered together (Fig. 6B).

**Table 2.** Inter- and intraspecific genetic differences among genus *Panelus* species for *COI* (658 bp) calculated using *p*-distance.

	***P. jinilli* sp. nov. (*n* = 3)**	***P. virginalis* sp. nov. (*n* = 2)**	***P. parvulus* (*n* = 5)**	***P. ovatus* (*n* = 1)**
*P. jinilli* sp. nov. (*n* = 3)	0.003			
*P. virginalis* sp. nov. (*n* = 2)	0.147–0.148	0.001		
*P. parvulus* (*n* = 5)	0.144–0.152	0.133–0.141	0.001–0.021	
*P. ovatus* (*n* = 1)	0.055–0.057	0.126	0.137–0.146	–

In Scarabaeidae, presumed cases of parthenogenesis have been reported in the genus *Cyclocephala* Dejean (Dutrillaux et al. 2014), but robust confirmation of this reproductive mode remains difficult. In the present study, all examined specimens of Panelus (Panelus) virginalis sp. nov. were female, and this repeated observation may be consistent with parthenogenesis. However, the absence of males alone cannot be regarded as conclusive evidence because alternative explanations remain possible, including sex-specific differences in behavior, phenology, microhabitat use, or detectability during collection. A possible role of reproductive endosymbionts such as *Wolbachia* also cannot be excluded, although *Wolbachia*-induced parthenogenesis has not been generally demonstrated in beetles (Kajtoch 2022). Moreover, the currently available molecular evidence is too limited to test this hypothesis rigorously, and mitochondrial data alone are insufficient to establish the reproductive mode (Rodriguero et al. 2010). We therefore regard parthenogenesis in this species as a tentative hypothesis that will require broader population-level sampling, additional genetic data, and screening for endosymbiotic bacteria in future studies.

## Supplementary Material

XML Treatment for Panelus

XML Treatment for Panelus (Panelus) jinilli

XML Treatment for Panelus (Panelus) virginalis

## Additional information

### Artificial Intelligence (AI) use

The authors accept full responsibility for the content of the manuscript, including the disclosure of any use of AI.

No AI tools were used in the preparation of this manuscript.

### Author ORCIDs

J. Shim https://orcid.org/0000-0002-4670-5108

K.-J. Ahn https://orcid.org/0000-0003-4880-0931

J.-H. Song https://orcid.org/0000-0003-0317-1457

### Data availability

All of the data that support the findings of this study are available in the main text or Supplementary Information.

## Supplementary materials

Supplementary material 1 List of two Japanese *Panelus* species Data type xlsx Explanation note **table SS1**. List of two Japanese *Panelus* species, including type material and voucher specimens, depositories, label data, voucher numbers, and GenBank accession numbers. Asterisks indicate new addition. https://binary.pensoft.net/file/1775910This dataset is made available under the Open Database License (http://opendatacommons.org/licenses/odbl/1.0/). The Open Database License (ODbL) is a license agreement intended to allow users to freely share, modify, and use this Dataset while maintaining this same freedom for others, provided that the original source and author(s) are credited. Jaeil Shim, Jeong-Hun Song
